# SARS-CoV-2 Binding and Neutralization Properties of Peptides Derived from N-Terminus of Human ACE2

**DOI:** 10.3390/ijms24098269

**Published:** 2023-05-05

**Authors:** Irina V. Astrakhantseva, Alina E. Ershova, Sergei A. Chuvpilo, Natalia A. Kruglova, Aydar A. Ishmukhametov, Marina S. Drutskaya, Liubov I. Kozlovskaya, Sergei A. Nedospasov

**Affiliations:** 1 Division of Immunobiology and Biomedicine, Sirius University of Science and Technology, Sirius, Krasnodarsky Krai, 354349 Sochi, Russia; astrakhantsevairina@gmail.com (I.V.A.);; 2 Laboratory of Gene Therapy of Socially Significant Diseases, Center for Precision Genome Editing and Genetic Technologies for Biomedicine, Institute of Gene Biology, Russian Academy of Sciences, 119334 Moscow, Russia; 3 Department of Emerging and Reemerging Infections, Chumakov Scientific Center for Research and Development of Immune-and-Biological Products, Russian Academy of Sciences (Institute of Poliomyelitis), 108819 Moscow, Russia; 4 Institute for Translational Medicine and Biotechnology, Sechenov First Moscow State Medical University (Sechenov University), 119435 Moscow, Russia; 5 Laboratory of Molecular Mechanisms of Immunity, Engelhardt Institute of Molecular Biology, Russian Academy of Sciences, 119991 Moscow, Russia

**Keywords:** spike protein, decoy receptor, coronavirus variants, viral entry inhibition, human angiotensin-converting enzyme 2

## Abstract

The binding properties of synthetic and recombinant peptides derived from N-terminal part of ACE2, the main receptor for SARS-CoV-2, were evaluated. Additionally, the ability of these peptides to prevent virus entry in vitro was addressed using both pseudovirus particles decorated with the S protein, as well as through infection of Vero cells with live SARS-CoV-2 virus. Surprisingly, in spite of effective binding to S protein, all linear peptides of various lengths failed to neutralize the viral infection in vitro. However, the P1st peptide that was chemically “stapled” in order to stabilize its alpha-helical structure was able to interfere with virus entry into ACE2-expressing cells. Interestingly, this peptide also neutralized pseudovirus particles decorated with S protein derived from the Omicron BA.1 virus, in spite of variations in key amino acid residues contacting ACE2.

## 1. Introduction

SARS-CoV-2, a coronavirus causing COVID-19, uses human angiotensin-converting enzyme 2 (ACE2) for entry into epithelial and other cells [1,2]. The spike (S) protein of the original and emerging SARS-CoV-2 variants directly interacts with ACE2 through its receptor-binding domain (RBD). ACE2 is a type I transmembrane glycoprotein whose main physiological function is the conversion of vasoconstrictive and inflammatory peptide angiotensin-2 into angiotensin. ACE2 is expressed in many organs and cells, most notably by the pulmonary alveolar epithelial cells and enterocytes of the small intestine [3], the two major sites affected by the SARS-CoV-2 infection. The S protein is a heavily glycosylated [4] 180 to 200 kDa transmembrane type 1 fusion protein with its N-terminus located at the outer surface of the viral particle and short C-terminus buried in the inner membrane space [5,6] that is cleaved at two different sites. The furin protease targets the site located between S1 and S2 subunits of S protein, followed by the additional cleavage at the S2′ site by transmembrane serine proteases (TMPRSS2 and cathepsin L) during virus entry. Such a cleavage into S1 and S2 subunits is essential for the initiation of membrane fusion and virus entry into the cell.

The interface for S protein-ACE2 interaction was revealed through structural studies [7,8,9]. Interestingly, molecular modeling predicted a stronger interaction of the SARS-CoV-2 RBD with the ACE2 receptor, as compared to less pathogenic SARS-CoV. While the S protein undergoes multiple variations in the emerging virus variants due to mutations, RBD-specific sites of the host ACE2 remain unchanged [10].

In many ligand-receptor systems represented by interacting protein domains, a soluble part of the cellular membrane-associated receptor may serve as an inhibitor of effective binding and signaling [11,12,13,14]. For example, among cytokine receptor superfamilies there is a number of so-called decoy-receptors or molecular traps that may be encoded by the same or a separate gene [15,16]. In the case of S protein interactions with ACE2, it was already reported that a soluble part of ACE2 may serve as an inhibitor of virus entry, thus representing a potential prophylactic/therapeutic substance [17,18,19]. We hypothesized that shorter peptides derived from the N-terminus of ACE2 that contain residues known to contact RBD in the complex with S protein or purified RBD may also inhibit viral infection.

In this study, we evaluated binding properties of several peptides ranging from 6 to 32 aa within the N-terminus of ACE2, as well as of a longer recombinant 80 aa polypeptide. The same peptide set was also evaluated for its ability to interfere with virus entry in vitro using pseudovirus particles (PVP) [20,21] or a live SARS-CoV-2. Finally, we compared the binding and neutralization properties of a 32 aa peptide in which the alpha-helix was stabilized by introduction of covalent bonds (a “stapled” peptide).

## 2. Results

### 2.1. Selection of Peptides for This Study

**Figure 1:**
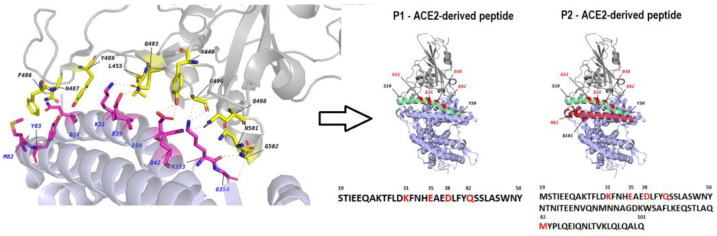
The interface of ACE2-Spike protein molecular interaction. (**Left**): ACE2 domain is depicted at the bottom (critical residues are colored in red), whereas the spike protein is depicted at the top (critical residues are in yellow). Nitrogen and oxygen in amino acid structure marked in blue and red, respectively. (**Right**): ACE-derived peptides P1 and P2. Helix I is colored in green, Helix II is colored in burgundy. Amino acid residues making direct contacts with RBD [22] are highlighted in red. 3D protein structure visualization was obtained using the PyMOL Molecular Graphics System (Section 4.6).

Molecular interface based on X-ray crystallography data that reveal the details of interaction between the S protein of SARS-CoV-2 virus (on the top) and ACE2 cellular receptor (on the bottom) is depicted in Figure 1. Several critical aa peptides of ACE2 that directly contact the RBD domain of the S protein reside within the alpha-helix located at the N-terminal region of ACE2.

Based on the map, we initially selected a P1 peptide that was composed of aa 19–50 within the N-terminal alpha-helix (Helix I) and included K31, E35, D38, and Q42 residues for further study (Figure 1). We also synthesized fluorescently labeled P1-FAM and mutated P1mut with alanine substitutions in four critical positions (Table 1).

In another study [23], several short ACE2 peptides (SAP) covering part of Helix 1 that included D38 and Q42 (Figure S1A) were evaluated with regard to their ACE2 binding. Some of these peptides, such as SAP1, SAP2, SAP6, and SAP-4 (the latter peptide was derived from another region of ACE-2 (Table 1), were also synthesized and used for comparison in our study.

**Table 1:** Amino acid sequence of ACE2 peptides which were used in the study.

Peptide	Amino Acid Sequence	Length, a.a.
P1	STIEEQAKTFLDKFNHEAEDLFYQSSLASWNY	32
P1m	STIEEQAKTFLD**A**FNH**A**AA**A**LF**A**QSSLASWNY ^1^	32
P1st	STIEEQAKT**X**LDK**X**NHEAEDLFYQ**X**SLA**X**WNY ^2^	32
P2 ^3^	MSTIEEQAKTFLDKFNHEAEDLFYQSSLASWNYNTNITEENVQNMNNAGDKWSAFLKEQSTLAQMYPLQEIQNLTVKLQLQALQ	80
SAP-1 ^4^	TFLDKFNHEAEDLFYQ	16
SAP-2 ^4^	EDLFYQSSL	9
SAP-4 ^4^	GKGDFRIL	8
SAP-6 ^4^	EDLFYQ	6

^1^ A—original amino acids in the P1 sequence are substituted with alanine; ^2^ X—(S)-2-(4-pentenyl)alanine (crosslinking agent); ^3^ described previously [24]; ^4^ described previously [23].

All chemically synthesized peptides were accompanied by carboxyfluorescein fluorophore (FAM)-containing versions that facilitated binding analyses.

Additionally, we evaluated properties of a longer peptide, P2, that comprised Helices I and II from the N-terminal part of ACE2. Helix II interacts with RBD via M82, and it could also stabilize the overall structure. Since chemical synthesis of such long peptides is challenging, this 80 aa peptide was produced using recombinant DNA technology [24]. Additionally, based on the study by Curreli et al. [25] the P1 peptide was modified so that Helix I could be stabilized by introducing covalent chemical crosslink (P1st, Table 1).

### 2.2. P1, Three SAP Peptides and P2 Can Specifically Bind to S Protein

**Figure 2:**
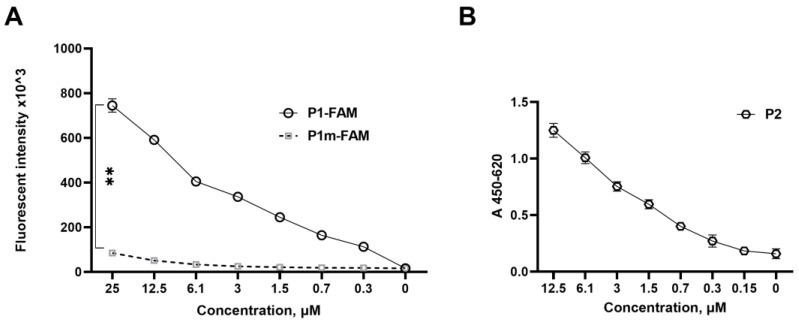
ACE2-derived peptides are able to bind S protein of SARS-CoV-2 (Wuhan variant). (**A**) P1 binds the S protein of SARS-CoV-2. Results of fluorescent immunoassay in which N-fluorescein FAM-labeled P1 was titrated on a microplate coated with full-length S protein of SARS-CoV-2. Mutant P1 (P1m) with alanine substitutions of key amino acids in positions 31, 35, 38, and 42 was used as a control. (**B**) P2 binding to full-length S protein of SARS-CoV-2 immobilized on high protein-binding plate was assessed by anti-P1 antibodies [24]. Data represent one of the three independent experiments. The data were analyzed using multiple unpaired *t*-test, **—*p* < 0.01.

We took advantage of fluorescent versions of synthetic peptides to directly evaluate their binding to immobilized full-length S protein (Figure 2A). As for the analysis of P2 binding, we used previously described highly specific anti-ACE2 antibodies [24] (Figure 2B). As expected, FAM-labeled P1m peptide failed to bind to immobilized S protein, while P1 and P2 demonstrated their binding ability (Figure 2A).

SAP proteins SAP1, SAP2, and the shortest SAP6 peptide showed reasonable binding, while SAP4 whose sequence was derived from Helix-11 failed to bind immobilized S protein (Figure S1B).

### 2.3. Neither P1 nor P2 or SAP Peptides Are Able to Interfere with the Virus Entry

**Figure 3:**
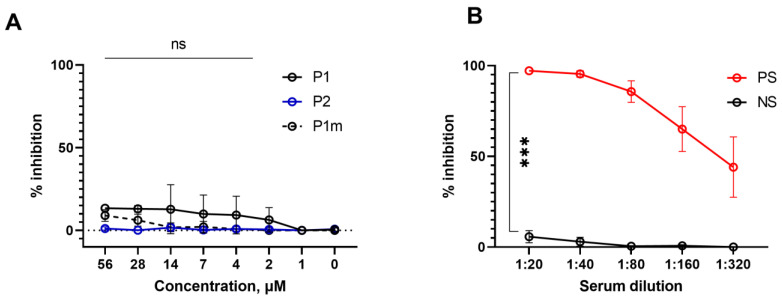
Linear peptides P1 and P2 could not inhibit entry of PVP with Wuhan variant S-protein into ACE2-overexpressing cells. (**A**) P1 and P2 were added in concentrations ranging from 0 to 56 μM to ACE-2-overexpressing HEK-293T cells exposed to (infected with) PVP. P1m was used as a control. (**B**) Serum from COVID-19 convalescent donors (PS) was used as a positive control, NS—negative sera for anti-SARS-CoV-2 antibodies. Data represent one of the three independent experiments. The data were analyzed using multiple unpaired *t*-test, ns—*p* > 0.05, ***—*p* < 0.001.

The main goal of our study was to identify peptides that could block the entry of SARS-CoV-2 into ACE2-expressing cells. To this end, we explored the artificial pseudovirus particle system based on a lentiviral platform that expressed S protein of the parental variant (Wuhan-1), as well as variants of concern (VOC) of SARS-CoV-2 [20,21]. PVPs employed in this study also contained expression plasmids for fluorescent protein GFP, allowing the quantification of the virus entry into ACE2-expressing HEK293 by flow cytometry. Surprisingly, none of the described peptides, including 80 aa-long P2 that demonstrated efficient binding to S protein or RBD, were able to inhibit PVP entry at the expected concentration range (Figure 3A). Positive and negative sera for anti-S antibodies were used as controls (Figure 3B).

Moreover, these experiments were also repeated with live SARS-CoV-2 virus infection of Vero cells with the same negative result (Figure S2). Some inhibition was observed with higher concentrations of SAP proteins; however, a non-binding peptide SAP4 showed the same low level of inhibition arguing against S-ACE2-mediated entry (Figure S3).

### 2.4. Stapled P1 Binds S Protein and Inhibits Virus Entry

**Figure 4:**
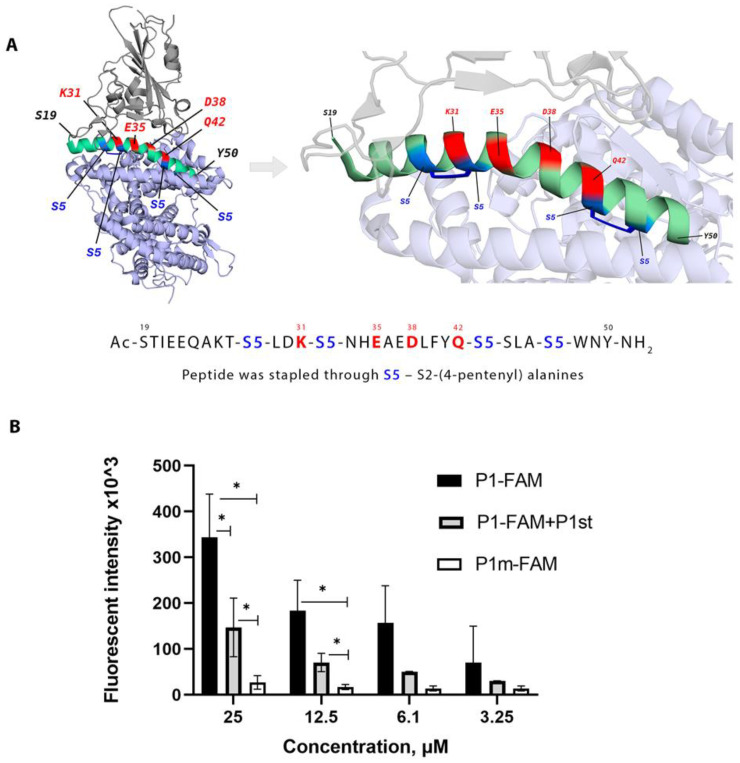
Properties of P1st stapled peptide. (**A**) Structure and aa sequence of P1 stapled peptide—P1st. Positions of substituted aa in Helix I are highlighted in blue; key aa positions for ACE2-S protein interaction are highlighted in red. 3D protein structure visualization was obtained using the PyMOL Molecular Graphics System (Section 4.6) (**B**) P1st peptide can compete with P1-FAM peptide for S protein binding. Axis X represents the concentrations of fluorescent P1-FAM (black) or P1m-FAM (white) ranging from 3.25 to 25 μM. P1st was added to P1-FAM (gray) at concentration of 25μM to compete for binding to S protein. Axis Y represents fluorescent intensity. Microplate was coated with full-length SARS-CoV-2 S protein (Wuhan variant), and binding of P1-FAM and P1m-FAM was assessed by fluorescent immunoassay. After incubation with peptides, fluorescence intensity values were measured at 483 nm excitation and 530 nm emission wavelengths. Data represent one of the three independent experiments. The data were analyzed using multiple unpaired *t*-test, *—*p* < 0.05.

Finally, we evaluated binding and inhibitory properties of the P1st peptide that contained four non-natural amino acids allowing subsequent highly specific covalent chemical crosslinking (Table 1, Figure 4A). Such “stapled” peptides were reported [26,27], and a similar but not identical to P1 peptide was evaluated in another study [25].

First, we established that P1st was able to efficiently compete with other peptides for binding to the S protein (Wuhan variant) (Figure 4B and Figure S1B).

**Figure 5:**
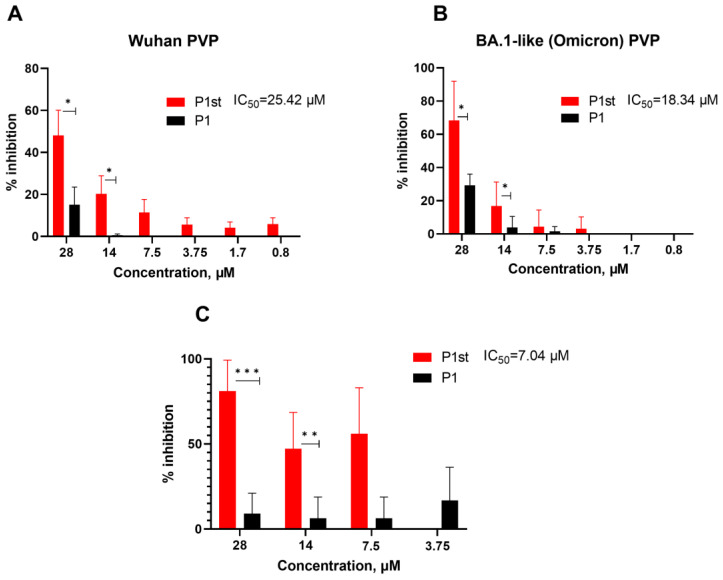
Effects of stapled and unstapled P1 peptide on PVP or SARS-CoV-2 entry in vitro. (**A**) Stapled peptide is able to effectively inhibit PVP entry mediated by Wuhan- (**B**) and BA.1-like (Omicron) variants of SARS-CoV-2 into ACE2-overexpressing cells, while an unstapled peptide does not. (**C**) Stapled peptide effectively inhibits infection of Vero cells with live SARS-CoV-2 virus (variant B.1.1), while an unstapled peptide does not. Data represent one of the three independent experiments. The data were analyzed using multiple unpaired *t*-test, *— *p<* 0.05, **—*p* < 0.01, ***—*p* < 0.001.

Importantly, P1st was able to inhibit the entry of Wuhan and BA.1-like S-protein-decorated PVPs and of live SARS-CoV-2 (B.1.1 variant) in a dose-dependent manner (Figure 5). Of particular interest is the fact that the pseudovirus system using the P1st peptide retained its inhibitory activity against more recent SARS-CoV-2 variants, such as Omicron BA.1-like (Figure 5B), in spite of the fact that amino acid residues in RBD contacting K31 and E35 и Q42 residues in ACE2 are mutated (Q493R and Q498R).

## 3. Discussion

This study sought to define minimal parts of soluble ACE2 that can bind S protein SARS-CoV-2 and interfere with virus entry into ACE2-expressing cells. Previously, Lei et al. showed that recombinant protein comprising a large portion of the extracellular domain of human ACE2 may serve as the efficient inhibitor of virus entry [28]. We initially selected the P1 32 aa-long peptide that comprised alpha Helix I known to directly interact with S protein through several critical amino acid residues [7]. This peptide showed effective binding to full-length S protein (Wuhan variant), but in the range of 0.8 to 28 micromoles failed to inhibit PVP entry into ACE2-overexpressing HEK293 cells (Figure 3 and Figure 5). A longer 80 aa P2 peptide that comprised both Helix I and Helix II and was making an additional contact with RBD (Figure 1) showed effective binding to S protein but also failed to inhibit PVP entry (for both Wuhan and BA.1-like S protein variants) (Figure 3). These negative results were reproduced in another in vitro system of infecting Vero cells with live SARS-CoV-2 virus (B.1.1 variant) (Figure S2). Our findings appear to contradict at least one study [23] claiming that even shorter SAP peptides with sequences derived from Helix I may bind RBD and efficiently interfere with virus entry. Several of such SAP peptides were synthesized and evaluated for binding and neutralizing efficiency. We were able to reproduce binding but not the inhibitory activity, as observed both for PVP-HEK293T/ACE2 and for SARS-CoV-2-Vero in vitro experimental systems (Figures S1 and S3). Thus, all examined peptides in their “linear” form at concentrations allowing binding to S protein were unable to serve as inhibitors of virus entry. Of note, at much higher concentrations all SAPs (including SAP4) showed inhibition of viral entry in spite the lack of SAP4 binding to immobilized S protein (Figure S3). We concluded from these experiments that the inhibition at higher concentrations is probably non-specific and maybe independent of S protein-ACE2 interaction.

Affinity of SARS-CoV-2 S protein binding to hACE2 relies on multiple atomic interactions that depend on the conformation of both proteins [29]. RBD makes contact with the N-terminal domain of hACE2 that includes several helices [30]. Short peptides are not able to form stable alpha-helices, and this may explain why they can bind to S protein in the solid phase setting (Figure 1 and Figure S1) but be ineffective in the neutralization reaction (Figure 3 and Figure S3). Additionally, short peptides are prone to degradation. There are several ways to increase stability of peptides including cyclization of linear peptides or introduction of stabilizing chemical bonds known as stapling [31]. Based on a previous study [25], we modified P1 peptide by covalent crosslinks based on (S)-2-(4-pentenyl)alanine (Table 1, Section 4.1).

Stapled P1 (P1st) peptide was able to compete with both P1 and SAP peptides for binding to S protein (Figure 4B and Figure S1). Of particular interest, only this peptide demonstrated dose-dependent inhibition of PVP entry into HEK293/ACE2 cells, as well as inhibition of in vitro infection of Vero 6 cells with live SARS-CoV2 (Figure 5). Moreover, P1st was equally effective in inhibiting entry of PVP decorated with Wuhan variant S protein as well as PVP with Omicron (BA.1) variant (Figure 5A,B). Somewhat surprisingly, in spite of a significant number of mutations within Omicron BA.1 S protein and its RBD, including mutations at positions Q493R and Q498R involved in the interaction with ACE2 (see Figure 1), these variations resulted in enhanced affinity of the S protein for the ACE2 receptor [32,33]. Of note, polymorphisms in human ACE2 that could potentially modulate ACE2-RBD interaction were reported, including the polymorphism in Helix I of ACE2 [34]. Thus, evaluation of peptides from such ACE2 variants for their ability to interact with RBD of emerging SARS-CoV-2 strains should be performed in the future.

## 4. Materials and Methods

### 4.1. Design of Experimental Peptides

The contact points between the Spike protein Receptor-binding domain (RBD) and ACE2 receptor [22,35] were used for to select a 32 aa-long peptide (designated P1) comprising sequences of Helix I at the N-terminus of ACE2 (19–50 aa). This peptide and a control 32 aa long peptide (designated P1m) with 3 mutations that were expected to significantly diminish its affinity to RBD were synthesized. SAP peptides with lengths of 6 to 16 aa described previously [23] were also synthetized. All peptides were produced using conventional solid phase (SPPS) technology with a Syro II automatic synthesizer (BCM Diagnostic, Woodland, CA, USA). To activate the protected amino acids, the DIPCDI/HOBt-a (Diisopropylcarbodiimide/Hydroxybenzotriazole-a) method was used, and N-terminal labeling of both peptides was performed by incorporating corresponding amino acid carrying FAM (5(6)-carboxyfluorescein) (Sigma, St. Louis, MO, USA). The stapled version of P1 peptide synthesis followed the standard solid-phase procedure. The peptidyl resin with full protection was used for cyclization with Grubbs first catalyst as described [25]. The peptide was cleaved from cyclized peptidyl resin, washed, and dried under vacuum overnight to give the crude stapled peptide. All peptides were purified through HPLC column (Sigma, St. Louis, MO, USA), and molecular weight was confirmed by mass spectrometry. Another ACE2-derived long peptide, designated as P2, comprised Helices I and II (see Figure 1) with aa sequences up to residue 101 of human ACE2 being expressed in *E. coli* and purified by standard methods [24].

### 4.2. Immunosorbent Assays

To quantify peptide binding to SARS-CoV-2, spike protein microplates were coated with recombinant full-length S protein (sequence derived from Wuhan-Hu-1 isolate) kindly provided by G. Efimov. Briefly, 100 μL of S protein at concentration 1 μg/mL in standard PBS buffer (pH 7.4, Sigma, St. Louis, MO, USA) was added per well to a 96-well microplate (FLUOTRAC™ 600 96W Microplate, Sigma, USA). The microplate was incubated overnight at 4 °C, then washed once with PBS with 0.05% Tween-20, and blocked by 5% BSA (Sigma, USA) in PBS for 2 h at RT. After two more washes, it was incubated with decreasing concentrations of the peptides conjugated with 5(6)-FAM for 2 h at RT followed by washing procedures. Fluorescence intensity values were measured at 483 nm excitation and 530 nm emission wavelengths (ClarioStar, Bmg Labtech, Ortenberg, Germany).

To assess P2 peptide binding to the S protein, microplates (MICROLON^®^ 200 96W Microplate, Sigma, USA) were coated with the recombinant full-length S protein as described above. Decreasing concentrations of P2 were added to the plate and incubated for 2 h at 37 °C. After 3 washings (0.05% Tween in PBS), aP1Ab was added as a primary antibody [24] at 1 μg/mL, incubated for 2 h at RT, and followed by 4 rounds of washings. Anti-rabbit IgG-HRP (ThermoFisher, Waltham, MA, USA) was used as a secondary antibody (1:40,000, for 1 h at RT followed by 5 rounds of washing). TMB (Sigma, USA) was used as a substrate (20 min at RT in the dark). The reaction was terminated by adding 0.2 M sulfuric acid. Optical absorbance (A450-620) was measured after P2 peptide dilutions were added to S protein-coated plate (Multiskan FC, ThermoFisher, USA).

### 4.3. Production of Pseudoviral Particles (PVP) Decorated with SARS-CoV-2 S Protein

To obtain PVP, HEK-293T cells (3 × 10^6^ cells) were plated on a 10 cm Petri dish and cultured overnight in 10 mL of the DMEM complete medium containing 10% FBS (HyClone, Logan, UT, USA), 2 mM L-glutamine, and penicillin/streptomycin at a concentration of 50 U/mL and 50 μg/mL, respectively (PanEco). The medium was replaced with DMEM containing 2 mM L-glutamine without FBS (10 mL). For transfection, 30 μL of an aqueous solution of polyethyleneimine (PEI, 1 mg/mL) and a solution of the plasmids pCMVdelta8.2R (5 μg), pUCHR-IR-GFP (6.67 μg), and pCG1-SARS-2S-deltaF-deltaC19 (3.33 μg) [1], containing a total of 15 μg DNA (DNA:PEI ratio = 1:2), were added to 1 mL of the OptiMEM culture medium (Capricon Scientiic GmbH, Ebsdorfergrund, Germany), and transfection was performed according to standard protocol [36]. After 48 h at 37 °C and 5% CO_2_, the supernatant was collected, centrifuged (2000× *g*, 4 °C, 5–7 min) to pellet any packaging cells or cellular debris cleared through 0.45 μm PES filters, and subjected to concentration through centrifugation (20,000× *g*, 4 °C, 2.5 h). The pellet containing PVP was resuspended in 1/20 of the original volume, divided into aliquots, and stored at −80 °C. PVP was titrated on ACE2-expressing HEK-293T and assessed by flow cytometry on a BD LSRFortessaTM analyzer (BD Biosciences, Franklin Lakes, NJ, USA). Dilutions of PVP at which viral transduction were between 20 and 30% were chosen for neutralization experiments.

### 4.4. Neutralization of PVP Transduction

This procedure was carried out as described previously [20], with minor modifications. For the neutralization experiment, ACE2-expressing HEK-293T were plated on 96-well TC-treated plate (Eppendorf, Hamburg, Germany) at 2 × 10^4^ cells/well. Non-immune human serum without antibodies against SARS-CoV-2 was used as a negative control, and the serum with a high antibody titer obtained in 2021 from a COVID-19 convalescent was used as a positive control. PVP were incubated with peptides in the concentration range from 0.8 to 30 μM in DMEM/F12 complete medium (PanEco, Moscow, Russia) for 60 min at room temperature and then added to the HEK-293T/ACE2 cell culture and incubated for 48 h at 37 °C and 5% CO_2_. Cells were carefully washed twice with PBS (pH 7.4). Then, CellWash buffer was added (1% BSA in PBS), and cells were detached from the plate by vigorous pipetting. Transduction analysis was performed using flow cytometry on a BD LSRFortessaTM analyzer (BD Biosciences, Franklin Lakes, NJ, USA) at the Resource Center of Cell Technology and Immunology of the Sirius University of Science and Technology.

### 4.5. Virus Neutralization Test

Vero cell line was obtained from Biologicals, World Health Organization, Switzerland (10-87). Cells were maintained in DMEM (Chumakov FSC R&D IBP RAS, Moscow, Russia), supplemented with 5% FBS (Gibco, New York, NY, USA), streptomycin (0.1 mg/mL), and penicillin (100 units/mL) (PanEco, Moscow, Russia). The SARS-CoV-2 strain PIK35 (Pango lineage B.1.1, GISAID EPI_ISL_428852) was used for experiments.

The efficiency infection inhibition was assessed as the peptide’s ability to decrease viral yields after one replication cycle. Virus was incubated with peptides in the concentration range from 0.8 to 30 μM in DMEM (Chumakov FSC R&D IBP RAS, Russia) for 60 min at 37 °C and 5% CO_2_ and then added to the Vero cell monolayers and incubated for 4 h 7 °C and 5% CO_2_ for non-neutralized virus adsorption and entry. Then, cells were washed from unsorbed virus and incubated for approx. 24 h at 37 °C and 5% CO_2_ for virus replication. Virus yields were determined via titration in Vero cells and RT-PCR (POLYVIR SARS-CoV-2, Lytech, Moscow, Russia). Inhibition was expressed as percent from untreated cells yields.

### 4.6. Data Analysis

Data analysis was performed using GraphPad Prism 9.5.0 software and presented as mean ± SEM. The comparative statistic was conducted using the unpaired *t*-test. Some 3D models of the proteins were made using the PyMOL Molecular Graphics System, version 2.5.4, PDB ID: 6VW1 (Schrödinger, LLC., New York, NY, USA). Flow cytometry data was processed using FlowJo 10.8.1 (BD, USA) and FlowAI 2.3.1 plugin [37].

## 5. Conclusions

ACE2 peptide comprising Helix I and stabilized by chemical modifications, known as staples, not only efficiently binds to RBD but inhibits virus entry to cells in vitro. Moreover, this inhibition is not limited to the earlier strain of SARS-CoV-2. However, current costs of stapled peptide synthesis may limit potential in vivo applications.

## Data Availability

The data presented in this study are available in this manuscript.

## Supplementary Materials

The following supporting information can be downloaded at: https://www.mdpi.com/article/10.3390/ijms24098269/s1.
