# Supplementary material for: SARS-CoV-2 Binding and Neutralization Properties of Peptides Derived from N-Terminus of Human ACE2
Source: Int J Mol Sci. 2023 May 5;24(9):8269. doi: 10.3390/ijms24098269 (PMC10179272; doi:10.3390/ijms24098269)
Supplement: Supplementary file 1 [file ijms-24-08269-s001.zip › ijms-2375639-supplementary.pdf]

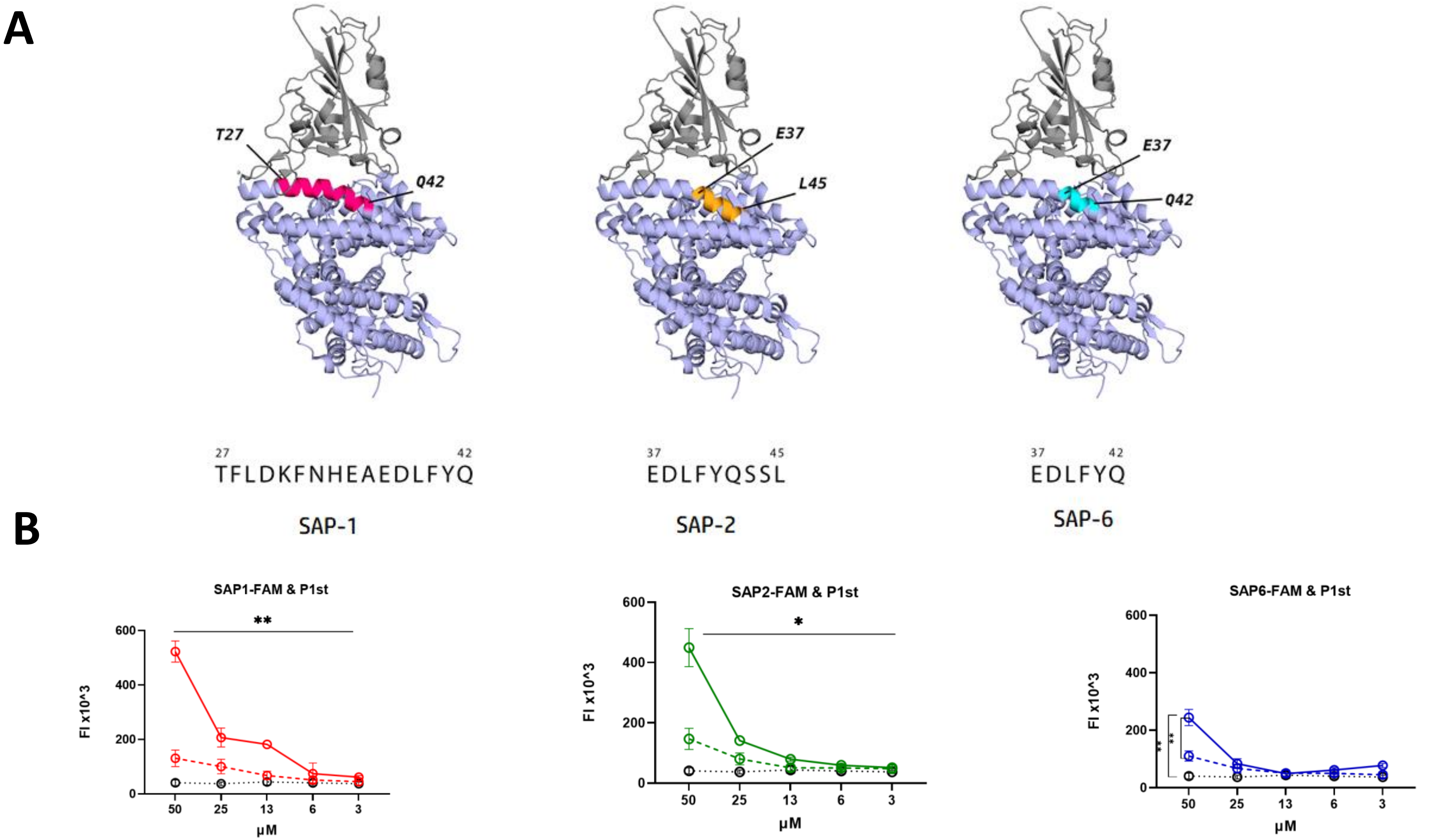

Figure S1. **Binding of short ACE2 peptides to S protein of Wuhan variant**.
(A) Localization of short ACE2 peptides at interaction interface. PDB ID: 6VW1 (https://www.rcsb.org/structure/6VW1)
(B) short peptides can bind S protein (solid colored line) while the stapled peptide (P1st) successfully competed with binding (dotted colored line). SAP-4 - GKGDFRIL was used as a control (solid black line). P1st concentration was 25μM. Axis shows concentrations of short peptides. Axis Y shows fluorescent intensity. Microplates were coated with full-length SARS-CoV-2 protein. After incubation with peptides fluorescent intensity values were measured at 483 nm excitation and 530 nm emission wavelengths. Fluorescent immunoassays were performed in triplicates, data represents one of the three independent experiments. The data were analyzed using multiple unpaired t-test, *p>0,05, **p>0,01

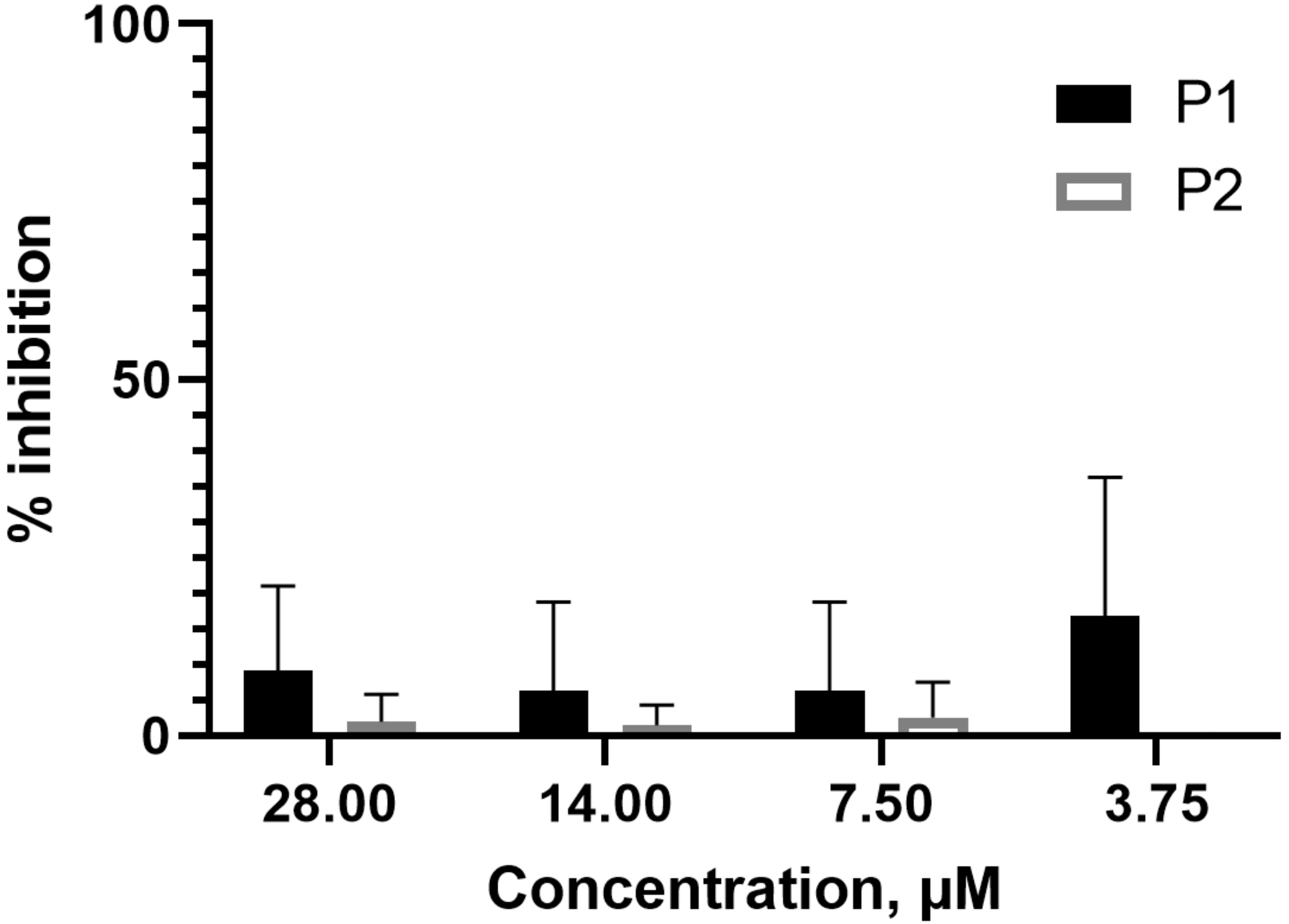

Figure S2. **Effects of linear peptides on SARS-CoV-2 (B.1.1) infection *in vitro*.**
P1 and P2 peptides do not inhibit SARs-CoV-2 infection in Vero cells. Data represents one of three independent experiments.

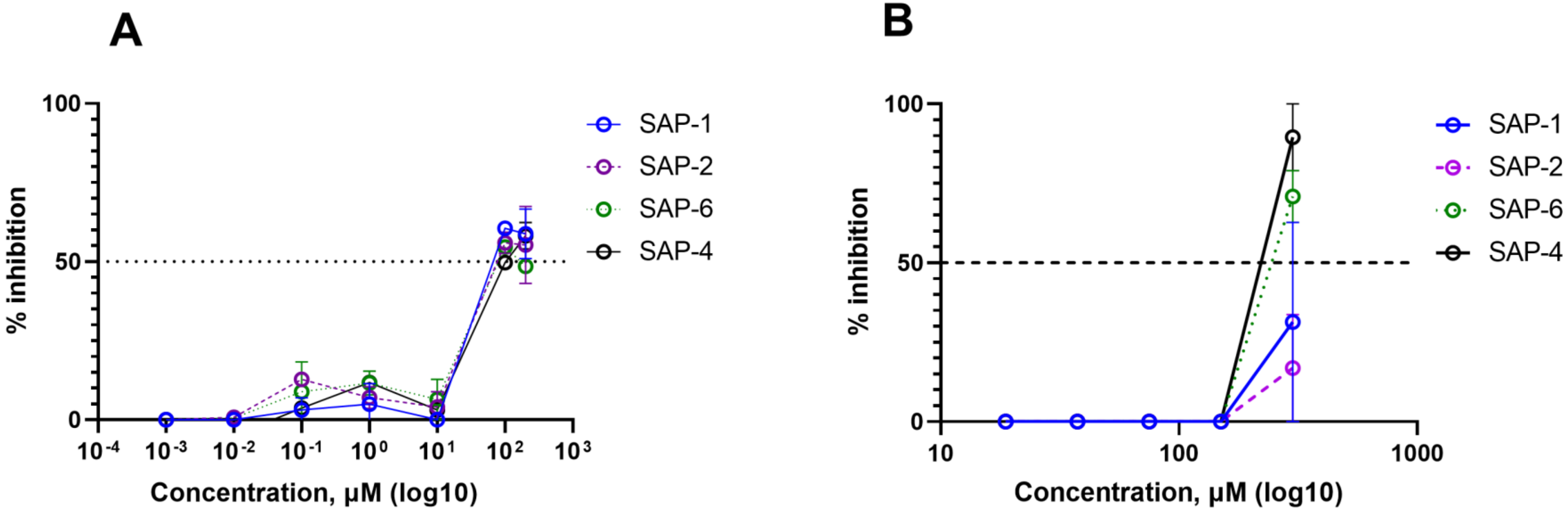

Figure S3. **Non-specific effects of short ACE-2 peptides (SAP) on PVP and virus entry into cells expressing ACE2.**
(A) PVPs containing Wuhan variant of the S protein were used with HEK/ACE2 cells. Marked inhibitory activity was observed at concentration of 200µM for all SAP peptides (including the control peptide SAP-4). (B) Live SARS-CoV-2 (B1.1 variant) infection of Vero cells. Inhibitory activity was observed at concentration of 300µM for all SAP peptides (including control peptide SAP-4).
